# Effects of mood episodes and comorbid anxiety on neuropsychological impairment in patients with bipolar spectrum disorder

**DOI:** 10.1002/brb3.1813

**Published:** 2020-08-30

**Authors:** Chih‐Chun Huang, Yun‐Hsuan Chang, Tzu‐Yun Wang, Sheng‐Yu Lee, Shiou‐Lan Chen, Po See Chen, Hsien‐Yuan Lane, Yen Kuang Yang, Ru‐Band Lu

**Affiliations:** ^1^ Department of Psychiatry Dou‐Liou Branch National Cheng Kung University Hospital Yunlin Taiwan; ^2^ Department of Psychology College of Medical and Health Science Asia University Taichung City Taiwan; ^3^ Department of Medical Research China Medical University Hospital Taichung City Taiwan; ^4^ Department of Psychiatry National Cheng Kung University Tainan City Taiwan; ^5^ Department of Psychiatry Kaohsiung Veteran's General Hospital Kaohsiung Taiwan; ^6^ Department of Psychiatry Faculty of Medicine Kaohsiung Medical University Kaohsiung Taiwan; ^7^ Graduate Institute of Medicine & M.Sc. Program in Tropical Medicine College of Medicine Kaohsiung Medical University (KMU) Kaohsiung Taiwan; ^8^ Department of Medical Research KMU Hospital Kaohsiung Taiwan; ^9^ Institute of Behavioral Medicine College of Medicine National Cheng Kung University Tainan Taiwan; ^10^ Department of Psychiatry and Brain Disease Research Center China Medical University Hospital Taichung Taiwan; ^11^ Graduate Institute of Biomedical Sciences China Medical University Taichung Taiwan; ^12^ Department of Psychiatry Tainan Hospital Ministry of Health and Welfare Tainan Taiwan; ^13^ Yanjiao Furen Hospital Hebei China

**Keywords:** anxiety disorder, bipolar disorder, comorbidity, neuropsychological impairment

## Abstract

**Objectives:**

Cases of patients with bipolar disorder (BD) having neuropsychological impairment have been reported, although inconsistently. The possibility of comorbidity with anxiety disorder (AD) has been suggested. The association between mood episodes and AD comorbidity on neuropsychological performance is unclear and thus was investigated in the current study.

**Methods:**

All participants were informed about and agreed to participate in this study. Patients with BD were recruited from outpatient and inpatient settings, and healthy controls (HCs) were recruited as a comparison group. Six hundred and twenty‐eight participants (175 HCs and 453 BD—56 BDI and 397 BDII) were studied based on their current mood episode, namely, depressive (BD_d_), manic/hypomanic (BDm), mixed (BDmix), and euthymic (BDeu), compared with/without AD comorbidity (164 with AD).

**Results:**

Compared to HCs, all BD groups had significantly more impaired neuropsychological profiles, but the BDeu group was found to have less impairment in memory and executive function than the episodic BD groups. The percentage of AD comorbidity in BDd, BDm, BDmix, and BDeu was 33.9%, 40.3%, 33.0%, and 35.6%, respectively (*χ* ^2^ = 1.61, *p* > .05). The results show that AD plays a different role in neuropsychological impairment across various mood episodes in BD.

**Conclusion:**

Memory impairment and executive dysfunction may be state‐like cognitive phenotypes and are affected by AD comorbidity during mixed and depressive episodes in BD, while sustained attention deficiencies are more like trait markers, regardless of mood episodes, and persist beyond the course of the illness. The AD comorbidity effect on attentional deficit is greater when suffering from a manic episode.

## 1 INTRODUCTION

It has been reported that pathological mood states affect cognitive performance in patients with bipolar disorder (BD). Cognitive changes in BD have been debated as transient and enduring (Clark & Goodwin, 2004). Reduced frontal cortex and hippocampus MRI volumes are correlated with sustained attentional impairment in manic BD patients (Sax et al., 1999). This implies a structural change and circulation between the inhibition and limbic systems, which involve emotional processes and memory functions. Impulsivity, another core feature frequently seen in BD patients, especially during manic episodes, is a multifaceted concept that encompasses a failure to inhibit inappropriate responses and poor judgments (Christodoulou, Lewis, Ploubidis, & Frangou, 2006; Strakowski et al., 2008). Cognition deficits in BD patients during manic episodes have been reported, and the impairment of impulse control has been found in a variety of experimental tasks, including in continuous performance tests (Clark & Goodwin, 2004). Impulsivity is also correlated with errors of commission in inhibitory control and flexibility tasks (Fino et al., 2014). A previous meta‐analysis suggested that possible deficits in the domains of executive function and memory may be candidate endophenotypes for BD patients (Bortolato, Miskowiak, Köhler, Vieta, & Carvalho, 2015). The connection between the impairment of executive function and the deficiency in impulse control implies that impulsivity and sustained attention deficits might be core impairments in BD (Glahn et al., 2010).

Although some aspects of neuropsychological impairment have been suggested as possible endophenotypes for identifying BD, inconsistent findings remain. The impairment in memory seems to have received the most agreement in previous literature (Bortolato et al., 2015; Chang et al., 2018; Ferrier, Stanton, Kelly, & Scott, 1999; Glahn et al., 2010; Goldberg & Burdick, 2008).

Taking mood episodes into consideration, broad cognitive impairment has been reported as being much severer in episodes of manic/mixed mood compared in euthymic (Sweeney, Kmiec, & Kupfer, 2000). In another study, the neuropsychological and psychosocial functions of 25 patients with bipolar I disorder (BDI) were assessed in a natural course of illness across several mood episodes (Malhi et al., 2007), and an association between neuropsychological and functional impairment with mood episode was found. They also reported that in euthymic BD, although characterized by an absence of symptoms, attention, and memory remained impaired. Latalova, Prasko, Pastucha, et al. (2011) further reported that in euthymic episodes, the subsequent cognitions, sustained attention, verbal memory, and executive functions were found to persist (Latalova, Prasko, Diveky, & Velartova, 2011; Latalova, Prasko, Pastucha, et al., 2011). To sum up, neuropsychological impairment in BD seems to persist, even in euthymic BD; however, whether these impairments are dynamically changed, namely, state‐like cognitive phenotypes in BD, is unclear. In addition, the impact of mania was previously reported in BD patients, but BD patients often suffer from mood swings containing depression, mania, and mixed episodes; however, the association between neuropsychological impairment and other mood episodes is unclear and needs further investigation.

Anxiety disorder (AD) has been reported as one of the most common comorbidities in BD, although the prevalence rate varies across different countries (Chang, Chen, et al., 2012; Nabavi, Mitchell, & Nutt, 2015; Spoorthy, Chakrabarti, & Grover, 2019; Yapici Eser, Kacar, Kilciksiz, Yalçinay‐Inan, & Ongur, 2018). The impact of comorbidity with AD in BD has drawn much attention, because more remarkable clinical characteristics have been noticed in BD patients with AD comorbidity, such as shortened periods of euthymia, prolonged treatment time to remission, and severer symptoms (Deckersbach et al., 2010; Spoorthy et al., 2019). In addition, AD comorbidity has been reported to worsen BD patients’ episodic symptoms and response to the treatment and to increase their suicidal behavior (Allen et al., 2005; Bauer et al., 2005; Simon et al., 2003, 2004). Moreover, an effect of AD comorbidity has been reported on neuropsychological impairment in BD (Chang, Chang, et al., 2012; Wu et al., 2011; Zutshi, Reddy, Thennarasu, & Chandrashekhar, 2006). As such, the importance of the effect of AD comorbidity on BD deserves more clinical attention and recognition (Perkonigg, Kessler, Storz, & Wittchen, 2000; Roy‐Byrne et al., 2000; Spoorthy et al., 2019). The profiles of the neuropsychological performance of BD patients with comorbidities (BD^+^) are varied, because wide domains of cognitive functions are disrupted (Bearden, Hoffman, & Cannon, 2001; Goldberg & Burdick, 2008; Quraishi & Frangou, 2002).

The impairments in memory in euthymic BD patients with AD comorbidity compared to those without AD have been reported (Wu et al., 2011), and the effect of AD on memory processing has been implied (Hua, Chang, Lin, Yang, & Chen, 2005). Moreover, the significant deficits in working and verbal memory suggest an impact on frontal lobe functioning, disruption of frontal–subcortical or mesolimbic circuitry, or partial executive dysfunction (Ferrier et al., 1999). Soraggi‐Frez, Santos, Albuquerque, and Malloy‐Diniz (2017) carried out a comprehensive review and suggested that working memory deficit is more likely to be state‐independent in BD, indicating that mood episodes are associated with working memory impairment (Soraggi‐Frez et al., 2017). Working memory is sometimes referred to be part of executive function and has been reported to be impaired in BD patients as well as in AD patients (Soraggi‐Frez et al., 2017). BD patients with AD comorbidity have been reported to have poor distractibility and worse attentiveness, but not necessarily poor vigilance (Lu & Chang, 2018), implying the impact of AD on attention. Psychophysiological evidence shows that early hypervigilance is highly associated with anxiety disorder, especially generalized anxiety disorder (GAD) (Weinberg & Hajcak, 2011). To summarize, BD patients suffer from mood changes, which has impact on their neuropsychological functioning, particularly for those with AD comorbidity.

Consistent findings regarding cognitive impairment have been reported in BD patients; however, whether the impairment remains across different mood episodes in BD patients did not draw much attention and received disagreement. In addition, there are not many studies of the effects of AD comorbidity on neuropsychological performance in BD. The various aspects of neuropsychological impairment may be mood‐related, and there might be an interaction between AD comorbidity and mood episodes on neuropsychological profiles in BD.

## 2 AIMS

The aims of this study were to compare the neuropsychological performance across various mood episodes in BD in order to find out which neuropsychological impairments are trait‐like and which are state‐like cognitive phenotypes in BD. In addition, whether or not there is an interaction between AD comorbidity and mood episodes on neuropsychological impairment was investigated. We first hypothesized that the deficits on neuropsychological impairment that persist across different mood episodes may represent as trait‐like cognitive profiles in BD. Moreover, we also hypothesized that AD comorbidity has an impact on neuropsychological impairment and is accompanied by mood episodes.

## 3 METHODS

### 3.1 Participants

This study protocol was approved by the Institutional Review Board for the Protection of Human Subjects at two medical centers. All participants were given information about the research and agreed to participate in this study by providing written informed consent.

Patients were recruited from outpatient or inpatient settings from two medical centers. Each participant was initially evaluated by a senior psychiatrist and then given a structured interview by a research team member using the Chinese version of the Modified Schedule of Affective Disorder and Schizophrenia‐ Life Time (SADS‐L) (Endicott & Spitzer, 1978), a good inter‐rater reliability, to ensure a diagnosis based on the Diagnostic and Statistical Manual of Mental Disorders, fifth edition (DSM‐5). Participants diagnosed with BD were recruited, and then, their symptoms and severity were evaluated using the Young Mania Rating Scale (YMRS) (Young, Biggs, Ziegler, & Meyer, 1978) and the Hamilton Depression Rating Scale (HDRS) (Hamilton, 1960, 1967).

The healthy controls (HCs) consisted of volunteers from the community who responded to the advertisements. The psychiatric conditions of each volunteer were screened using the Chinese version of the SADS‐L and recruit with a high risk of presenting major mental disorders, who had a family history of psychiatric disorder among their first‐degree relatives, and those with a history of major mental disorders were excluded. In addition, both patients and HCs with other medical conditions and alcohol use disorders that may affect cognitive functions were excluded.

### 3.2 Neuropsychological tasks

#### 3.2.1 Continuous performance test

The Conners' Continuous Performance Test (CPT) (Conners & Staff, 2000) has been widely used to measure the maintenance of focused attention and the capability to inhibit impulsive responses, in order to combine some level of executive control so as to inhibit target‐resembling stimuli. In reports of the CPT, four capabilities of attention are categorized, namely, inattentiveness, impulsivity, sustained attention, and vigilance. Based on our assumptions, the following indices were chosen for further comparison: (1) inattentiveness—the measurement indices include (a) errors of omission (i.e., incorrect responses to the target), (b) detectability (*d*´; i.e., the capability to discriminate between targets and nontargets), and (c) variability, which represents the consistency in the speed of responses; (2) impulsivity, represented by the hit reaction time (HRT), which is the mean response time (milliseconds) for all target responses over the full trial; (3) sustained attention, measured by the HRT using block changes and by changes in reaction time across the duration of the test where high scores indicate a substantial slowing in reaction times; and (4) vigilance, measured based on the average RT across different HRT interstimulus intervals (HRT ISI Changes).

#### 3.2.2 Wisconsin Card Sorting Test

The Wisconsin Card Sorting Test (WCST) measures executive functions, mainly testing the functions of shifting and strategic planning (Heaton, Chelune, Talley, Kay, & Gurtiss, 1993). Fino et al. (2014) claim that the indices, number of errors, perseverative errors, and nonperseverative errors measure the inhibition ability of executive functions (Fino et al., 2014). Some components of cognitive function have been claimed to be requirements of performing well on the WCST, for example, attention, working memory, and visual processing. The WCST has an inter‐rater reliability of 0.88–0.93 and a test–retest reliability of 0.57. The WCST performance scores were based on the total number of errors (TNEs), perseverative errors (PEs), conceptual level responses (CLRs), number of categories completed (NCCs), and number of trials needed to complete the first category (TCCs).

#### 3.2.3 Wechsler Memory Scale, third edition

The Wechsler Memory Scale, third edition (WMS‐III) (Wechsler & Stone, 1997), the most frequently used memory function set, produces eight composite index scores covering immediate and delayed recognition and recall of both auditory and visual stimuli. Eight standardized indices are calculated and scored: Auditory Immediate (AIM), Visual Immediate (VIM), Immediate Memory (IM), Auditory Delayed (ADM), Visual Delayed (VDM), Auditory Delayed Recognition (ADRM), General Memory (GM), and Working Memory (WM).

### 3.3 Statistics

The chi‐square test was used to examine differences related to sex and other categorical variables. Because not all variables were normally distributed, nonparametric analyses, namely, Mann–Whitney *U* tests, were used to compare the differences between the HCs and the BD groups and between BD^+AD^ and BD^−AD^ across various mood episodes in BD. The Kruskal–Wallis test was used for comparisons between the HCs and the BD groups across various mood episodes, and the Dunn test was subsequently used for post hoc analyses (IBM SPSS 22.0, Armonk, NY, USA).

## 4 RESULTS

We recruited 628 participants: 175 HCs and 453 BD patients (56 BDI and 397 BDII). The HCs were significantly younger than those in the BD groups (*Z* = –2.40, *p* = .02) and had higher levels of education (Z = 7.25, *p* < .0005). Moreover, there was a significantly higher percentage of females in the BD group compared to in the HC group (*χ* ^2^ = 8.97, *p* = .003). In the power analysis, the effect size conventions were determined as follows: For the chi‐square test, a small effect size of 0.10, a medium effect size of 0.30, and a large effect size of 0.50; and for the mixed model with four groups, a small effect size of 0.10, a medium effect size**•** of 0.25, and a large effect size of 0.40 (Buchner, Erdfelder, & Faul, 1996). The numbers in our study (HCs versus BD patients = 175 versus 453) reached a large effect size and had a power of approximately 0.8. For the two group comparisons using the Mann–Whitney *U* test, our sample for each group (HC, BDd, BDm, BDeu, and BDmix = 173, 56, 134, 113, and 149, respectively) also reached a large effect size. For the effect of AD comorbidity, the numbers in each group (BDd^±AD^, BDm^±AD^, BDeu^±AD^, and BDmix^±AD^) and the 18 variables from the different domains of cognitive functions, a medium to large effect was reached.

For the comparisons of neuropsychological performance, the results showed that the BD groups generally had poorer performance than the HCs (Table 1).

**TABLE 1:** Distribution of the demographic characteristics in normal healthy controls (HCs) and in patients with bipolar disorder (BD)

Variables	HCs	BD patients	*Z*/*χ* ^2^ (*p*)
Gender (male/female)	99/76	196/257	8.97 (.003)
Age (mean ± *SD*) (median)	31.20 ± 8.25 (29.00)	35.64 ± 13.27 (33.49)	–2.41 (.02)
Educational level (years)	15.34 ± 1.71 (16.0)	13.50 ± 3.21 (14.0)	–7.21 (<.0005)
Age at onset of disorder	—	15.17 ± 5.04 (14)	—
Duration of illness	—	19.31 ± 12.83 (16)	—
Hamilton Depression Rating Scale Score	—	9.29 ± 57.41 (13)	—
Young Mania Rating Scale Score	—	8.21 ± 6.18 (12.5)	—
Continuous Performance Test (CPT) (*n* = 172 versus 423)			
Omission T‐score	47.22 ± 12.80 (44.87)	92.55 ± 89.78 (54.66)	–9.94 (<.0005)
Commission T‐score	48.60 ± 10.53 (46.94)	55.55 ± 12.30 (54.31)	–6.45 (<.0005)
HRT T‐score	45.73 ± 9.56 (43.96)	53.91 ± 14.76 (51.92)	–7.30 (<.0005)
Variability T‐score	44.93 ± 9.96 (43.08)	60.19 ± 18.12 (56.15)	–10.21 (<.0005)
Detectability (*d*,) T‐score	48.69 ± 9.92 (49.39)	53.51 ± 9.88 (54.36)	–5.27 (<.0005)
HRT Block Change T‐score	50.69 ± 8.73 (49.64)	55.85 ± 14.53 (53.93)	–4.18 (<.0005)
HRT ISI Change T‐score	49.57 ± 9.30 (49.57)	54.12 ± 37.94 (50.42)	–2.13 (.03)
Wisconsin Card Sorting Test (WCST) (*n* = 175 versus 453)			
Total number of correct_ T (TNC)	95.39 ± 18.55 (103)	90.09 ± 19.87 (95)	–3.86 (<.0005)
Total number of errors_ T (TNE)	31.51 ± 16.95 (25)	36.98 ± 18.82 (32)	–4.01 (<.0005)
Nonperseveration response_T (NPE)	14.07 ± 8.57 (12)	16.02 ± 9.35 (14)	–2.71 (.007)
Perseveration error_T (PE)	18.42 ± 14.61 (13)	21.19 ± 15.41 (16)	–3.76 (<.0005)
Number of completed categories_T (NCC)	7.43 ± 2.42 (8)	6.12 ± 2.85 (7)	–5.36 (<.0005)
Trials to complete the first category_T (TCC)	16.38 ± 11.90 (12)	17.14 ± 13.53 (12)	–0.03 (.98)
Wechsler Memory Scale, third edition (WMS‐III) (*n* = 173 versus 375)			
Auditory immediate (AIM)	101.34 ± 25.94 (105)	101.45 ± 33.38 (100)	–3.44 (.001)
Visual immediate (VIM)	94.23 ± 25.87 (100)	97.05 ± 17.22 (97)	–1.04 (.30)
Immediate memory (IM)	100.46 ± 20.23 (104)	98.41 ± 17.20 (98)	–3.08 (.002)
Auditory delayed (ADM)	101.27 ± 25.30 (108)	99.21 ± 18.00 (102)	–3.68 (<.0005)
Visual delayed (VDM)	93.98 ± 24.88 (100)	96.30 ± 18.28 (97)	–0.53 (.59)
Auditory recognition delayed (ARDM)	100.49 ± 28.46 (110)	98.39 ± 16.69 (100)	–4.07 (<.0005)
General memory (GM)	101.67 ± 18.07 (104)	97.74 ± 18.02 (98)	–3.33 (.001)
Working memory (WM)	103.20 ± 20.62 (106)	96.07 ± 15.19 (97)	–6.46 (<.0005)

To investigate whether the neuropsychological impairment in BD would be affected by mood episodes, BD patients with HDRS_17_ ≤ 12 and YMRS ≤ 10 were considered euthymic in this study based on previous suggestion (Altinay, Hulvershorn, Karne, Beall, & Anand, 2016). All BD patients were categorized into four subgroups on the basis of their mood conditions when they entered this study, namely, BDd, BDm, BDmix, and BDeu. Overall, the BD groups had neuropsychological impairment regardless of their mood episodes compared to the HCs. The BD euthymic (BDeu) group had less impairment in some aspects of cognition compared with the other BD groups (Table 2). For the attention domain, the significantly higher rate of target missing and the greater variability in episodic BD (BDd, BDm, and BDmix) than in euthymic BD (BDeu) imply that BD patients have inattentiveness during mood episodes. In addition, those with episodic BD (BDm and BDmix) were found to have higher impulsivity and vigilance. For the executive functioning domain, the BDd and BDm groups were found to have more perseverative errors compared to the BDeu group, implying that BD patients often engage in perseveration during depressive and manic/hypomanic episodes. For the memory domain, no significant differences in any memory indices were found between episodic BD and euthymic BD. In addition, no significant differences were found between BDeu and HCs, except in the working memory index (WMI).

**TABLE 2:** Comparisons of scores on the neuropsychological battery in BD patients among different mood states

	Healthy control (HC; *n* = 175)	BD patients				*χ* ^2^ (*p* value)	Post hoc
		Depressive (BDd; *n* = 56)	Manic/hypomanic (BDm; *n* = 134)	Mixed‐episode (BDmix; *n* = 149)	Euthymic (BDeu; *n* = 113)		
Sex (male/female)	99/76	24/32	65/69	57/92	49/64	12.12 (.02)	—
Age (mean ± *SD*)	31.20 ± 8.25 (29.00)	35.80 ± 13.26	35.98 ± 14.15	35.91 ± 12.86	34.75 ± 12.81	6.48 (.26)	—
AD comorbidity rate (*n*, %)	—	19 (33.3)	54 (40.3)	53 (35.6)	37 (33.3)	1.69 (.64)	—
Educational level (years)	15.34 ± 1.71 (16.0)	13.86 ± 2.80	13.45 ± 3.20	13.22 ± 3.24	13.85 ± 3.33	55.20 (<.0005)	^b^HC > BDd; ^b^HC > BDm; ^b^HC > BDmix; ^b^HC > BDeu
Age at onset of disorder	—	15.12 ± 5.26	14.42 ± 4.13	14.78 ± 4.36	16.57 ± 6.38	14.49 (.002)	^b^BDeu > BDm; ^a^BDeu > BDmix
Duration of illness	—	18.90 ± 11.59	20.50 ± 13.81	20.18 ± 12.79	16.76 ± 11.20	5.52 (.14)	—
Hamilton Depression Rating Scale Score	—	18.52 ± 2.20	11.12 ± 2.52	17.49 ± 2.08	6.02 ± 3.03	—	—
Young Mania^c^ Rating Scale Score	—	8.02 ± 1.97	14.43 ± 1.59	13.66 ± 2.45	6.39 ± 2.50	—	—
Continuous Performance Test (CPT)							
Omission_T	47.22 ± 12.80 (44.87)	81.64 ± 67.84 (56.51)	93.26 ± 79.75 (58.42)	93.26 ± 79.75 (58.42)	66.71 ± 63.84 (46.62)	141.15 (<.0005)	^b^HC < BDd; ^b^HC < BDm; HC < BDmix; ^a^HC < BDeu; ^a^BDeu < BDd; ^c^BDeu < BDm; ^c^BDeu < BDmix
HRT_T	45.73 ± 9.56 (43.96)	53.92 ± 13.80 (54.80)	54.18 ± 14.99 (52.44)	56.54 ± 15.34 (52.44)	49.39 ± 12.45 (48.36)	65.91 (<.0005)	^c^HC < BDd; ^c^HC < BDm; ^c^HC > BDmix; ^a^HC < BDeu; ^c^BDeu < BDmix
Variability_T	44.93 ± 9.96 (43.08)	60.19 ± 14.74 (57.46)	62.88 ± 17.88 (59.53)	62.88 ± 17.88 (59.53)	50.59 ± 15.99 (46.88)	150.55 (<0.0005)	^b^HC > BDmix; ^a^HC < BDeu; ^b^BDeu < BDd; ^c^BDeu < BDm; ^c^BDeu < BDmix
D’	48.69 ± 9.92 (49.39)	54.60 ± 8.00 (54.88)	55.48 ± 9.22 (56.63)	55.47 ± 9.22 (56.63)	50.16 ± 10.60 (50.19)	47.30 (<0.0005)	^c^HC < BDm; ^c^HC < BDmix; ^b^HC < BDd; ^b^BDeu < BDm; ^a^BDeu < BDmix
HRT Block_T	49.57 ± 9.30 (49.57)	53.89 ± 16.20 (51.97)	57.15 ± 14.23 (53.92)	53.89 ± 16.20 (51.97)	52.65 ± 13.43 (51.98)	27.07 (<0.0005)	^b^HC < BDm; ^c^HC < BDmix; ^a^BDeu < BDmix
Wisconsin Card Sorting Test (WCST)							
Total number of correct_ T (TNC)	95.39 ± 18.55 (103)	87.05 ± 20.69 (91)	89.04 ± 18.58 (93.50)	89.04 ± 18.58 (93.50)	94.68 ± 17.46 (100.00)	25.38 (<.0005)	^a^HC > BDd; ^b^HC > BDm; ^b^HC > BDmix
Total number of errors_ T (TNE)	31.51 ± 16.95 (25.00)	39.48 ± 18.96 (37.00)	38.77 ± 18.43 (34.50)	38.77 ± 18.43 (34.50)	32.95 ± 16.72 (28.00)	25.82 (<.0005)	^a^HC < BDd; ^c^HC < BDm; ^a^HC < BDmix; ^a^BDeu < BDm
Perseveration errors_ T (PE)	18.42 ± 14.61 (13.00)	22.38 ± 12.99 (20.00)	23.55 ± 17.24 (18.50)	23.55 ± 17.24 (18.50)	17.54 ± 11.83 (13.00)	31.71 (<.0005)	^b^HC < BDd; ^b^HC < BDm; ^a^BDeu < BDd; ^b^BDeu < BDm
Number of completed categories_ T (NCC)	7.43 ± 2.42 (8.00)	5.36 ± 3.18 (6.00)	5.96 ± 2.68 (6.00)	5.96 ± 2.68 (6.00)	6.81 ± 2.83 (7.00)	40.75 (<0.0005)	^c^HC > BDd; ^c^HC > BDm; ^c^HC > BDmix; ^a^BDeu > BDd
Trials to complete the first category_ T (TCC)	16.38 ± 11.90 (12.00)	18.55 ± 15.12 (13.00)	17.70 ± 14.18 (12.00)	17.70 ± 14.18 (12.00)	17.53 ± 15.38 (12.00)	2.10 (.72)	—
Wechsler Memory Scale, third edition (WMS‐III)							
Auditory Immediate (AIM)	101.34 ± 25.94 (105)	110.27 ± 87.25 (97.00)	99.33 ± 16.72 (100.00)	99.33 ± 16.72 (100.00)	103.78 ± 19.17 (105.00)	16.85 (.002)	^a^HC > BDm; ^a^HC > BDmix
Visual Immediate (VIM)	94.23 ± 25.87 (100.00)	95.14 ± 16.28 (94.00)	95.11 ± 16.56 (97.00)	95.11 ± 16.56 (97.00)	102.41 ± 16.83 (10.00)	8.00 (.09)	—
Immediate Memory (IM)	100.46 ± 20.23 (104.00)	95.88 ± 15.41 (96.00)	96.96 ± 16.31 (98.00)	96.96 ± 16.31 (98.00)	103.60 ± 18.14 (103.00)	17.10 (.002)	^a^HC > BDd
Auditory Delayed (ADM)	101.27 ± 25.30 (108.00)	96.76 ± 15.90 (94.00)	99.02 ± 18.07 (105.00)	99.02 ± 18.07 (105.00)	104.07 ± 19.23 (105.00)	22.50 (<.0005)	^a^HC > BDd; ^b^HC > BDmix
Visual Delayed (VDM)	93.98 ± 24.88 (100.00)	93.27 ± 16.73 (94.00)	95.42 ± 16.77 (100.00)	95.42 ± 16.77 (100.00)	101.61 ± 19.19 (97.00)	6.52 (.16)	—
Auditory Recognition Delayed (ARDM)	100.49 ± 28.46 (110.00)	98.16 ± 17.52 (100.00)	97.48 ± 15.93 (100.00)	97.48 ± 15.93 (100.00)	101.85 ± 16.51 (100.00)	19.81 (.001)	^a^HC > BDm; ^b^HC > BDmix
General Memory (GM)	101.67 ± 18.07 (104.00)	94.73 ± 16.27 (94.00)	96.78 ± 17.08 (101.00)	96.78 ± 17.08 (101.00)	103.52 ± 19.69 (102.00)	20.86 (<.0005)	^a^HC > BDd; ^a^HC > BDm; ^a^HC > BDmix
Working Memory (WM)	103.20 ± 20.62 (106.00)	96.42 ± 16.51 (97.00)	96.27 ± 14.93 (97.00)	96.27 ± 14.93 (97.00)	98.52 ± 17.28 (100.00)	46.80 (<.0005)	^a^HC > BDd; ^a^HC > BDeu; ^c^HC > BDm; ^c^HC > BDmix

Note:

Data represented as mean ± standard deviation (*SD*) (median); the direction of arrow (i.e., < or>) represents better performance; ^a^ *p* < .05; ^b^ *p* < .005; ^c^ *p* < .0001 (considering statistical corrections).

**Figure 1:**
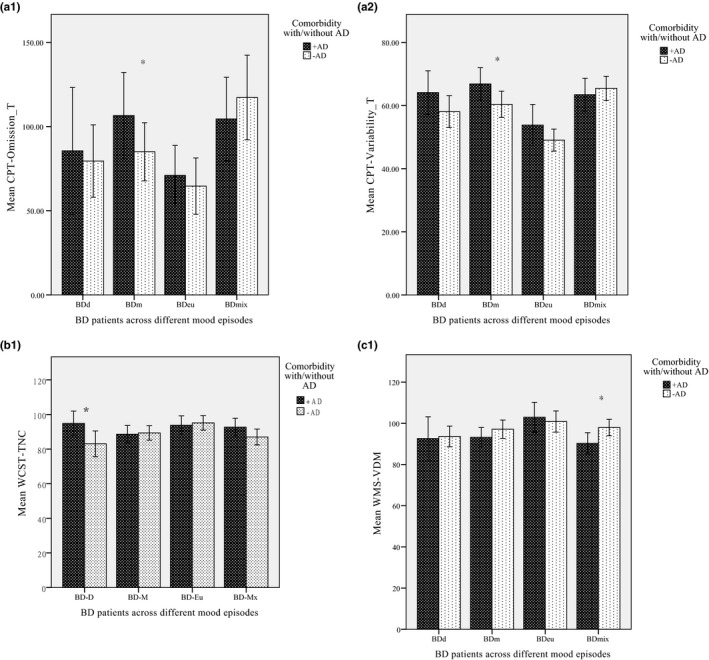
The influence of anxiety disorder (AD) comorbidity on various subscales of neuropsychological function in bipolar disorder (BD) (a‐1~c‐1)

The percentage of AD comorbidity in BDd, BDm, BDmix, and BDeu was 33.9%, 40.3%, 33.0%, and 35.6%, respectively (*χ* ^2^ = 1.61, *p* > .05). Because not all variables were normally distributed, nonparametric analyses were used to compare the effects of AD comorbidity across different mood episodes. The Mann–Whitney *U* tests showed that in the BDm group, those with AD comorbidity (BDm^+AD^) were found to have a significantly higher rate of target missing and variability in reacting to the performance than those with BDm^−AD^ (*Z* = –2.29 and *Z* = –2.13, respectively) (Figure 1a‐1,a‐2). In addition, in the BDd group, the AD comorbidity group (BDd^+AD^) had significantly lower accuracy in the WCST (*Z* = –2.00, *p* < .005) (Figure 1b‐1). For the comparisons of memory, those with BD and AD comorbidity in the mixed condition (BDmix^+AD^) had significantly worse performance in visual delayed memory compared to the BDmix^−AD^ group (*Z* = –2.36, *p* < .005) (Figure 1c‐1).

Generalized anxiety disorder (GAD) was a major subtype of AD comorbidity among BD^+AD^ (63.7%) in the current study; the numbers of other AD comorbidities were small. We further looked into the effect of GAD on the neuropsychological performance in BD^+AD^ across various mood states. We found an effect of GAD on executive function impairment in the BDmix^+AD^ group (BD^+GAD^ versus BD^−GAD^ = 29 versus 17), including number of errors and perseverative errors (*Z* = –2.29 and *Z* = –2.5, *p* < .05, respectively).

## 5 DISCUSSION

Bipolar spectrum disorders have been categorized as major mental disorders with mood swings that result in neuropsychological impairment and functional deficiencies. However, whether neuropsychological impairment is a mood‐related or a trait‐like phenotype remains unclear. The results in the current study showed that patients with BD had significantly poorer performance across a variety of neuropsychological domains than did the HCs, regardless of the mood episodes.

The findings in the current study showed that BD patients have poorer attention, which is in agreement with previous findings (Maalouf et al., 2010). Moreover, regardless of the mood episodes, impairment in sustained attention was found in all BD groups, including the BDeu group, implying that impairment on sustained attention may persist beyond the disorder (Ferrier et al., 1999; Malhi et al., 2007).

Moreover, compared to HCs, the BD groups were found to have impairments in memory functioning across various mood episodes, but no significant difference was found between the BDeu group and the HCs. In addition, no significant difference was found between the BDd and BDm groups, which is inconsistent with previous findings (Murphy et al., 1999). Our findings imply that memory impairment could be a mood state‐like profile for the prognosis of BD.

In terms of executive impairment, the results showed significant differences between euthymic BD (i.e., BDeu) and episodic BD (i.e., BDd, BDm, and BDmix), but no significant difference was found between the HCs and the BDeu group. The BDeu group showed similar performance compared to the HCs, indicating that executive impairment might be recoverable or could be considered as an indicator while BD patients are remitted from mood disturbance (Levy, Manove, & Weiss, 2012).

The effect of AD comorbidity has been reported to lead to neuropsychological impairment in BD (C.‐T. Chang, Chang, et al., 2012; Wu et al., 2011; Zutshi et al., 2006). According to the DSM‐5 and prior findings, AD might impair BD patients’ attention, might make them more vigilant, and might raise their arousal level; anxiety could be classed as a symptom of BD (APA, 2013). Poorer concentration and memory and difficulty in making decisions are often seen in BD, as well as in patients with anxiety disorders, which might explain the high comorbidity rate of anxiety and depression, as BD patients tend to suffer longer depressive episodes (Hirschfeld, 2001; Lamers et al., 2011; Wu & Fang, 2014). The results in the current study showed that the BDd^+AD^ group had significantly higher accuracy in the WCST than did the BDd^−AD^. This is in disagreement with a previous report of executive dysfunction in depressed patients with AD comorbidity (Basso et al., 2007) and may represent a difference in clinical characteristics between unipolar depression and bipolar depression (Taylor Tavares et al., 2007), thus warranting further investigation.

The subtypes and severity of AD were not measured in the current study, which might limit the generalizability of our findings. Previous investigations have reported different domains of memory impairment between BDII with and without AD comorbidity, but not between BDI with and without AD comorbidity (Chang, Chang, et al., 2012; Ferrier et al., 1999; Wu et al., 2011). The severity of neuropsychological impairment in BDII patients has been shown to be intermediate and between that of BDI patients and HCs (Hsiao et al., 2009; Torrent et al., 2006), whereas other researchers have reported that euthymic BDII patients have more severe impairment than do euthymic BDI patients and HCs (Harkavy‐Friedman et al., 2006; Simonsen et al., 2008; Summers, Papadopoulou, Bruno, Cipolotti, & Ron, 2006). Although this inconsistency has been suggested to be an impact of AD comorbidity on different subtypes of BD, the influence of AD on neuropsychological impairment across various mood episodes between subtypes of BD needs further investigation with a larger sample population.

Simeonova et al. (2009) reported that BD patients with AD comorbidity have a smaller hippocampal volume than those without AD comorbidity, which may be caused by longstanding anxiety‐induced overactivity of the hypothalamic–pituitary–adrenocortical axis and consequent impairments of the hippocampus (Simeonova et al., 2009). Consequently, AD comorbidity in BD patients may appear to result in not only neurocognitive dysfunctions, but also in persistent neurobiological lesions of the brain.

### 5.1 Limitations

This study has some limitations: First of all, not all participants performed all neuropsychological tasks. The completion rate for all tasks was as follows: 71.93% in the BDd group, 64.18% in the BDm group, 57.05% in the BDmix group, and 73% in the BDeu group. The effect of mood swings in BD needs further investigation with a longitudinal follow‐up design. In addition, no significant difference in memory impairment was found between the BDd and BDm groups; a possible explanation may be that the bias in selective attention could be mood‐related, but the abilities measured by WMS are more general. Moreover, the effect of AD subtypes on neuropsychological impairment in BD was not examined in the current study. Although GAD was found to be the major comorbidity subtype in the current study, similarly to a previous report (Chang, Chen, et al., 2012), it has been suggested that GAD may be associated with BD for classified diagnosis (Simon et al., 2003). A larger sample and a more specific categorized AD comorbidity with BD are needed in further investigations. Moreover, the severity of patients’ anxiety was not evaluated, which may have affected the performance of the BD patients in the neuropsychological tasks. A longitudinal design is needed for the follow‐up to study how AD affects the neuropsychological profile of BD patients across different mood episodes.

## 6 CONCLUSION

Bipolar disorder is characterized by mood dysregulation and causes widespread neuropsychological impairment. The impact of pathological mood episodes on neuropsychological impairment was found to be less important than that of AD comorbidity. In addition, an effect of AD comorbidity on different aspects of cognitive impairment was found. Further investigation on the interaction between mood episodes and AD comorbidity is urgently needed using a longitudinal study design.

## CONFLICT OF INTEREST

The authors declare no conflicts of interests.

## AUTHORS’ CONTRIBUTIONS

Y.H.C. performed the statistical analyses and wrote the first draft with H.C.C. The co‐authors T.Y.W., S.Y.L., P.S.C., Y.K.Y., H.Y.L., and R.B.L. managed the patient recruitment and assessment, as well as the literature review. All authors contributed to and approved the final version of the manuscript.

## ETHICAL APPROVAL

This study protocol was approved by the Institutional Review Board for the Protection of Human Subjects at National Cheng Kung University Hospital (IRB#A‐BR‐103‐078, IRB#A‐BR‐104‐089, and IRB#A‐BR‐108‐001) and China Medical University Hospital (IRB# CMUH106‐REC3‐023 and IRB#CMUH108‐REC3‐024). All participants were given information about the research and agreed to participate in this study by providing written informed consent.

### Peer Review

The peer review history for this article is available at https://publons.com/publon/10.1002/brb3.1813.

## Data Availability

The data that support the findings of this study are available on request from the corresponding author. The data are not publicly available due to privacy or ethical restrictions.
